# High rate capability caused by surface cubic spinels in Li-rich layer-structured cathodes for Li-ion batteries

**DOI:** 10.1038/srep03094

**Published:** 2013-10-31

**Authors:** Bohang Song, Hongwei Liu, Zongwen Liu, Pengfei Xiao, Man On Lai, Li Lu

**Affiliations:** 1 Materials Science Group, Department of Mechanical Engineering National University of Singapore, Singapore; 2 School of Chemical and Bimolecular Engineering The University of Sydney, Australia

## Abstract

Modified Li-rich layered cathode Li(Li_0.2_Mn_0.54_Ni_0.13_Co_0.13_)O_2_ has been synthesized by a simple strategy of using surface treatment with various amounts (0–30 wt.%) of Super P (carbon black). Based on detailed characterizations from X-ray diffraction (XRD), high resolution transmission electron microscope (HRTEM), X-ray photoelectron spectroscopy (XPS) and electrochemical impedance spectroscopy (EIS), it is suggested that the phase transformation from Li_2_MnO_3_-type of structure to spinel-like phase take place at the surface regions of particles during post annealing process at 350°C, leading to increase in both first coulombic efficiency and rate capability, from 78% and 100 mAh·g^−1^ (charge capacity at 2500 mA·g^−1^) of the pristine material to 93.4% and 200 mAh·g^−1^. The evidences of spinel formation and the reasons for electrochemical enhancement are systematically investigated.

To overcome the critical drawbacks of commercial used LiCoO_2_ such as limited energy density, high cost, toxicity and etc., an alternative cathode within layered structure is urgently needed to meet rapid development of hybrid electric vehicles (HEV) and electric vehicles (EV)^1^,^2^,^3^. To this end, the composite integrated in a nano-scale in form of layered-layered xLi_2_MnO_3_·(1-x)LiMO_2_ (M refers to commonly-used transition metals) has become appealing in a recent decade because the applicable specific capacity in such unique framework can reach as high as 250 mAh·g^−1^ 4,5. This is close to the theoretical capacity of layered cathodes. The high reversible capacity is believed to be associated with an initial loss of oxygen from the lattice as well as a corresponding phase transition in the first charging process^6^,^7^,^8^,^9^. However, the essential reasons for the structural stability at a deep delithiated state compared to other layered members such as Li_1-x_CoO_2_ and Li_1-x_MnO_2_ (0 < x < 1) are still not clear. Thackeray et al.^4^ claimed that the substitution of units (Li_2_MnO_3_) rather than cations or anions in the structure is the main reason for the substantial stability. In this regard, the control of activated amounts of Li_2_MnO_3_ during first charging process is critical for following reversibility but limits the usage of real capacity^10^. On the other hand, although the group of Li-rich cathode materials is easy to achieve high reversible capacity, large irreversible capacity in the first cycle as well as the poor rate capability still gap them from the real applications.

To understand principles and mechanisms governed poor rate capability, some research groups have used advanced techniques to lead investigation. Using real time gas analysis, Hong et al.^11^ observed that the oxygen ions are reversibly involved in the formation of Li_2_CO_3_ species through surface regions of particles after the first activation, which could contribute to reversible capacity. However, such kinetic process during formation and decomposition of Li_2_CO_3_ may slow down the flow rate of both ions and electrons which could result in poor rate capability during cycling. Using high-resolution scanning transmission electron microscopy (STEM), Gu et al.^12^,^13^ discovered that the nickel ions prefer to be selectively segregated at the surface facets of Li(Li_0.2_Ni_0.2_Mn_0.6_)O_2_ particles during high temperature synthesis driven by thermodynamic force. As a result, this aggregation causes negative impacts on rate performance of this cathode material because the fast diffusion channels of Li within these surface planes are almost perpendicular to the fast ion diffusion channels inside particle, leading to an inconsecutive pathway for fast Li ion transportation. Moreover, Koga et al.^14^ and Sathiya et al.^15^ have confirmed a reversible O^2−^/O^−^ redox process in bulk regions of Li-rich layered particles, whereas surface oxygen is oxidized to O_2_ and irreversibly lost during first charge, leading to transition metals migration and partial densification at particle surfaces.

As a matter of fact, all the discoveries mentioned above imply that such group of Li and Mn-rich cathode materials is surface-sensitive in consideration of electrochemical properties, of particular rate capability^16^,^17^. Therefore, a plenty of works have been done to improve the properties by using surface modification strategy. For instance, Li-Ni-PO_4_ compositional compound has been proven to be effective as a protection layer for high potential ranges, meanwhile it also contributes to enhance rate capability because of its excellent feature as Li ion conductor^18^,^19^. On the other hand, MnO_x_ nano-layer coating has also been applied to this Li-rich system to approach higher rate and specific capacity. One of the possible reasons for the enhancement could be the spinel-phase formation during the post-annealing process led by reintercalation of Li into lowly-crystallized MnO_x_ layers^20^,^21^. As another route to achieve the formation of surface spinel in this layered xLi_2_MnO_3_·(1-x)LiMO_2_ compounds, an acidic environment is required when modifying the surface of particles. It is widely accepted that the acidic environment in H_2_SO_4_ or HNO_3_ aqueous solution could result in proton exchange reaction between H^+^ and Li^+^, leading to Li vacancies in layered framework^10^,^22^. The similar observations in both (NH_4_)_2_SO_4_^23^ and AlF_3_^24^ surface treatments further induce that the post-annealing process following H^+^/Li^+^ exchange reaction is necessary to lead to spinel transformation in surface regions of Li_2_MnO_3_-contained layered particles. One of the possible reasons could be the instability of Li_2-x_H_x_MnO_3_ where the transformation tends to take place at a high temperature.

In the present work, carbon black (Super P) is used to modify Li(Li_0.2_Mn_0.54_Ni_0.13_Co_0.13_)O_2_ (LLNCM) particle surfaces. It has been found that the rate capability as well as cyclability of the modified sample is significantly improved. Most surprisingly, the carbon source is reasonably believed to contribute to spinel formation in our case based on careful investigations led by XRD, XPS, HRTEM, and electrochemical characterizations.

## Results

### Crystallographic and morphology of pristine and modified LLNCM

**Figure 1:**
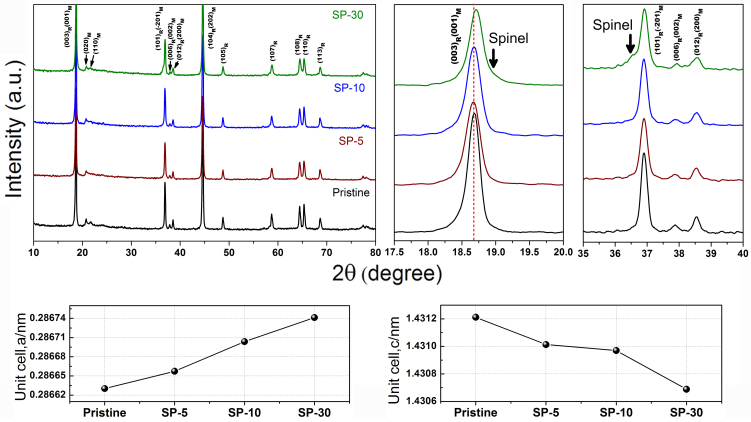
Powder XRD patterns of pristine, SP-5, SP-10 and SP-30 samples with a comparison of corresponding lattice parameters obtained by Rietveld refinement.

Fig. 1 shows the powder XRD patterns of the LLNCM before and after surface treatment using various amounts of Super P. Although all the strong peaks could be indexed to a rhombohedral phase with R-3m symmetry which is normally taken as the signature of LiMO_2_ phase, several weak peaks located in the range of 20–23°are consistent with the LiMn_6_ super-ordering in Li_2_MnO_3_ monoclinic phase with C2/m symmetry, as distinguished by “R” and “M” subscripts in Fig. 1. Detailed comparison of peak change or shift could be found in the selective patterns in the range of 17.5–20° and 35–40°. Compared to the pristine sample, the main (003)_R_/(001)_M_ peak slightly shifts to a higher degree in the case of SP-30 while the peaks for SP-5 and SP-10 samples barely move. On the other hand, two clear humps besides (003)_R_ and (101)_R_ peaks are easily identified in SP-30 sample, which is reasonably deduced as an evidence of spinel formation because similar features of peak shoulders emerged in the layered-layered-spinel Li_x_Mn_0.65_Ni_0.35_O_y_ system^25^ and Li_1 + x_(Ni_0.25_Co_0.125_Mn_0.625_)O_2.19 + x/2_ system^26^. However, it is hard to conclude a comparable spinel formation in the case of both SP-5 and SP-10 samples just according to XRD patterns. Therefore, Rietveld refinements within R-3m phase were conducted using General Structure Analysis System (GSAS) software^27^ to indicate the variations from the view of lattice parameters. The refinement results are summarized in Table 1. The lattice constant *a* in the unit cell slightly increases from 2.8663 (pristine) to 2.8674 Å (SP-30) through 2.8666 (SP-5) and 2.8670 Å (SP-10). On the contrary, the lattice constant *c* gradually decreases from 14.3121 (pristine) to 14.3069 Å (SP-30) through 14.3101 (SP-5) and 14.3097 Å (SP-10). The trends of lattice constant evolution are represented also in Fig. 1. Such consecutive changes in the unit cell of layered lattice are believed to be associated with the surface treatment led by Super P, furthermore with an increasing amount of formation of spinel-like ordering phase or increment of oxygen vacancy. Because analogous variation trends in lattice constants in case of acid-treated 0.5Li_2_MnO_3_·0.5LiNi_0.44_Co_0.25_Mn_0.31_O_2_ were previously observed^22^, indicating that both acid and Super P treatment possibly lead to comparable consequences of spinel formation after the post annealing process in this layered-layered system. In comparison, only heat treatment at 350°C without Super P is not able to transform the structure for P-350 sample, which could be supported from XRD pattern in Fig. S1.

**Figure 2:**
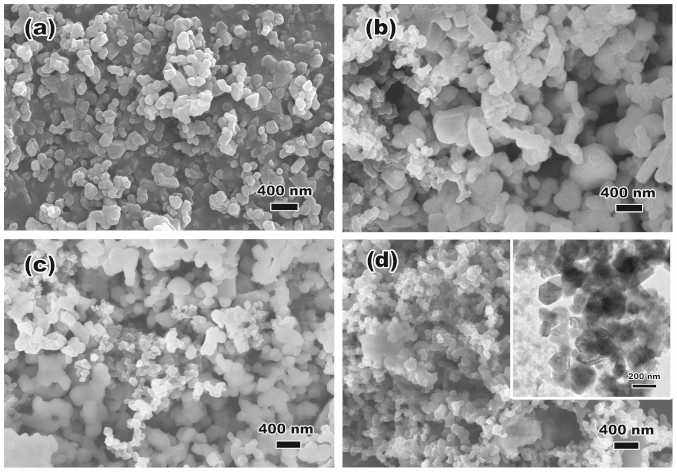
SEM and TEM images of (a) pristine, (b) SP-5, (c) SP-10 and (d) SP-30 powders. The brighter spheres with smaller particle size compared to LLNCM are residual Super P particles after post-annealing process at 350°C.

The morphologies of the LLNCM powders before and after surface treatment are compared in Fig. 2. The uniform distribution of highly-crystallized particles which are about 100 nm could be observed from Fig. 2(a). After surface treatment, not only the modified LLNCM particles but also a certain amount of carbon spheres with brighter appearance are easily to be identified in all the SP-5 (Fig. 2(b)), SP-10 (Fig. 2(c)) and SP-30 (Fig. 2(d)) samples. Not surprisingly, since the post annealing temperature applied to all the samples are 350°C, it led to only partial oxidation of carbon black in the air atmosphere from the blends which has been confirmed by TGA analysis (Fig. S2). According to TGA results, the pristine material shows 0.88% weight loss during heating from room temperature to 800°C, indicating a slight amount of evaporation of the adsorbed H_2_O molecules as well as oxygen loss from layered structure at high temperature. For all the treated samples, not only these effects but also the residual Super P causes weight loss from composite structure. Eliminating both of the adsorbed H_2_O and oxygen loss effects, SP-5, SP-10 and SP-30 possess 0.45, 1.85 and 12.7% of weight of residual Super P.

**Figure 3:**
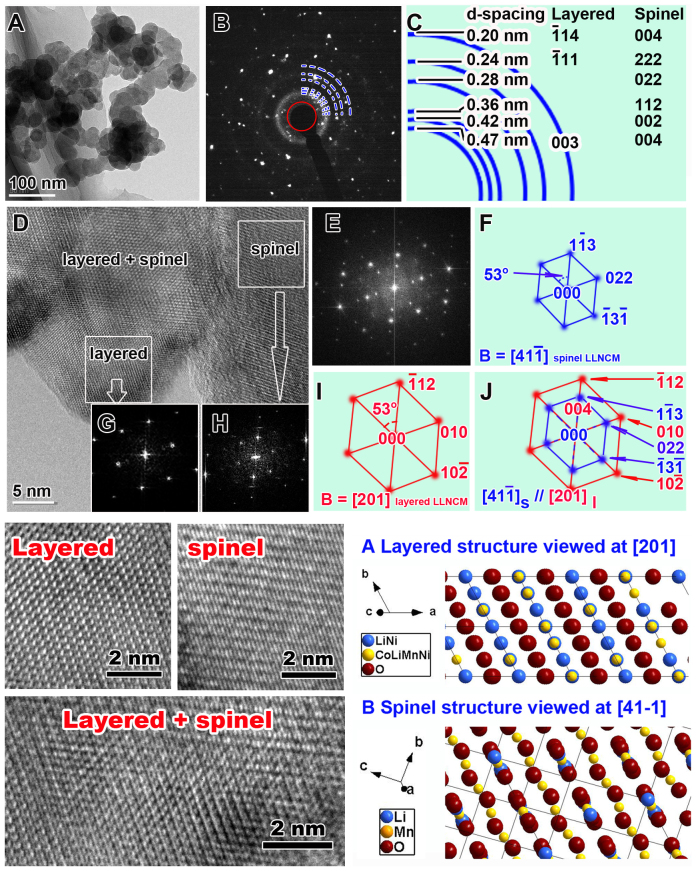
TEM identification of SP-30 particles. A. Low magnification TEM bright field image. B. Electron diffraction pattern corresponding to Panel A. C. The index to the electron diffraction rings in Panel B which could tell two kinds of LLNCM phases, i.e. layered monoclinic structure and cubic spinel structure. D. HRTEM image at surface regions shows layered, spinel and layered-spinel intermediate nano-domain structures. The fast flourier transformation (FFT) to Panel D is shown in Panel E which is indexed as in Pane J. The parallel zone axis for them are [201] *_layered_* and [41-1]*_spinel_*. Further FFT images for sub-areas as shown in Panel G and H are indexed to Panel I and F, respectively. Magnifications of layered, spinel, and layered-spinel intermediate zones obtained from Panel D compared with the simulated projections of atomic configurations of these two phases are also shown.

HRTEM was also employed to investigate the atomic changes of the SP-30 powder after surface treatment. As shown in Fig. 3A, the low magnification TEM bright field image not only reveals the aggregation effects between the LLNCM particles and carbon spheres, but also shows smooth facets of crystallized LLNCM particles with smaller particle size compared to the pristine sample (Fig. S3). The electron diffraction pattern corresponding to Fig. 3A is shown in Fig. 3B, while the index to these scattered diffraction spots is represented in Fig. 3C. Accordingly, it could be concluded that two phases are composed, which are layered monoclinic structure with parameters a = b = 0.284 nm, c = 1.44 nm, beta = 101.16° and newly-formed spinel cubic structure with parameter a = 0.83 nm. Furthermore, in view of the surface areas shown in HRTEM image of panel D, the original layered, the newly-formed spinel as well as the layered-spinel intermediate zone, all within nano-domains in one particle could be easily identified. The Fast Fourier Transformation (FFT) to Panel D is shown in Panel E which is indexed as in Panel J. The zone axis applied for them are [201]*_layered_* and [41-1]*_spinel_*. Further FFT images with corresponding indexing results for sub-areas are shown in panel G and H along with I and F, respectively, indicating the spatial relationship among these nano-domains. Some regions preserve layered ordering while others transform themselves into spinel ordering, meanwhile the regions are intermediate zones with both integrated orderings. A close contact between the LLNCM particles and carbon spheres after mixing is reasonable to be responsible for such partial transformation. Another interesting observation is that the similarity in symmetry between the two structures of layered and transformed spinel is obvious, as indicated in Fig. 3J. In other words, these two phases share a similar two-fold symmetry at this parallel orientation, which could be easily identified when comparing the magnified HRTEM images of different phase zones with the respective projections of atomic configurations in Fig. 3. Furthermore, it is reasonable to deduce that such highly-ordered integrated LiMO_2_-LiM_2_O_4_ (M refers to Mn, Co and Ni) phase is derived from original LiMO_2_-Li_2_MO_3_ phase as a result of Super P treatment, because the structural compatibility in view of (003)_trigonal_ and (001)_monoclinic_ lattice fringes is widely accepted in LiMO_2_-Li_2_MO_3_ compound^4^. In fact, similar transformation from layered Li_2_MO_3_ to spinel-like LiM_2_O_4_ phase due to a high temperature treatment has been reported by Boulineau et al.^28^, which confirms the possibility of phase transformation between these two close phases. It is also important to note that the only difference between SP-30 and other treated samples should be incremental areas of transformed spinels on particle surfaces rather than incremental depths of spinels into a single particle. Because adding more Super P only means that more surface areas of LLNCM particle could be contacted by carbon spheres, while the transformation effect on depth or structure has no change.

### Electrochemical performance of pristine and modified LLNCM

**Figure 4:**
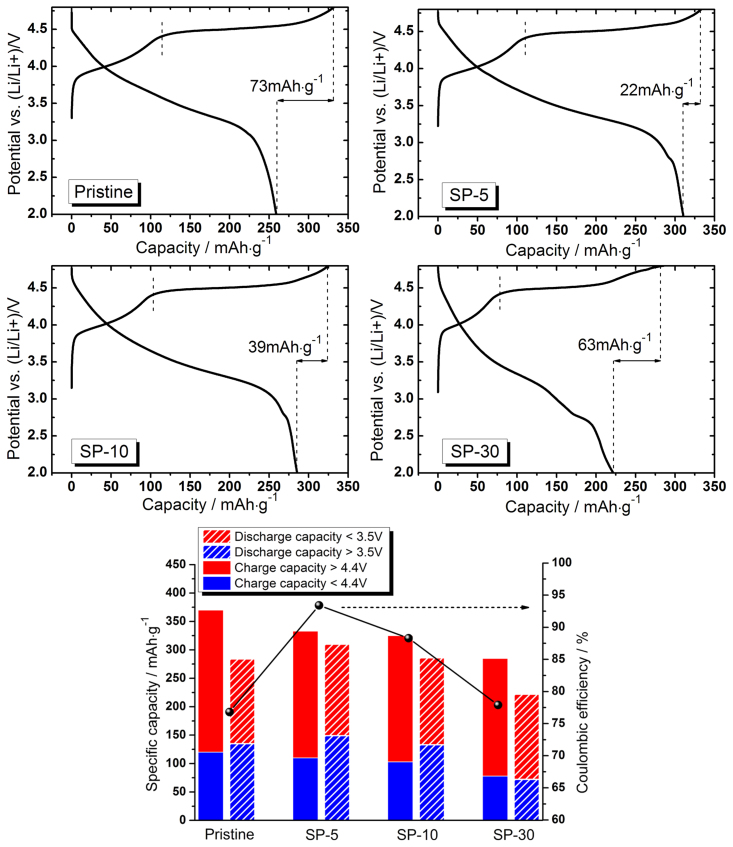
First charge/discharge curves with coulombic efficiency of pristine, SP-5, SP-10 and SP-30 cathodes when cycled between 2.0 and 4.8 V at 12.5 mA·g^−1^ (C/20).

The first charge/discharge curves during electrochemical cycling of corresponding cathode materials are shown in Fig. 4. At a low rate of 12.5 mA·g^−1^, the pristine LLNCM is able to delivery 260 mAh·g^−1^ discharge capacity. Surprisingly, the SP-5 and SP-10 cathodes are capable of providing 311 and 286 mAh·g^−1^ discharge capacities with increased first coulombic efficiencies from 78% (pristine) to 93.4% (SP-5) and 88.3% (SP-10). However, both of the discharge capacity and coulombic efficiency, i.e. 222 mAh·g^−1^ and 77.9% become worse than the pristine one with respect to SP-30. The reason could be ascribed to the weight contribution (12.7%) of residual Super P in SP-30 sample while they are electrochemically-inactive. If the weight impacts from residual Super P are eliminated, the discharge capacities for SP-5, SP-10 and SP-30 could be recalculated to 312, 292 and 255 mAh·g^−1^ respectively. It confirms that the LLNCM powder treated by 5% Super P delivers the highest capacity compared to the pristine and other treated samples. It is interesting to note from the histogram in Fig. 4 that the discharge capacity contributions from lower 3.5 V part with respect to SP-5 and SP-10 are higher than the counterpart of the pristine, which is reasonably believed to be associated with more active redox of Mn^4+^/Mn^(4-x)+^ reaction^5^. Correspondingly, the charge capacity contributions from higher 4.4 V part in both SP-5 and SP-10 samples are lower than the counterpart of the pristine, which could be understood by partial loss of active Li_2_MnO_3_ phase during the treatment. Combined with another abnormal observation of a newly-formed 2.7 V plateau in all the modified samples which is frequently recognized as a signal of spinel formation^22^,^24^,^26^, the increased discharge capacity as well as the enhanced first coulombic efficiency is reasonably explained by the phase transformation from Li_2_MnO_3_-active to corresponding spinel-active component, leading to a shortened 4.5 V charge plateau but an extended 2.7 V discharge plateau in all the modified samples. In comparison, an absence of 2.7 V discharge plateau for P-350 sample (Fig. S4) confirms no spinels after heat treatment without Super P. Note that such hypothetical mechanism of transformation is different from the one to explain voltage decay upon cycling because the former one is based on reduction effect from carbon, whereas the later one is based on Li vacancy and migration of transition metals upon cycling. The obtained values in consideration of the first charge/discharge cycle are also summarized in Table 2. In addition, a first discharge test from fresh state of new electrode was also performed for all samples. The results are shown in Fig. S5. It can be seen that the treated samples were able to exhibit 9, 12 and 46 mAh·g^−1^ discharge capacities for SP-5, SP-10 and SP-30. It indicates that the amount of transformed spinel phases is highly associated with the applied amount of Super P as the treatment agent. In contrast, the well crystallized particle in layered form of pristine LLNCM provided no capacity when discharged to 2.0 V.

**Figure 5:**
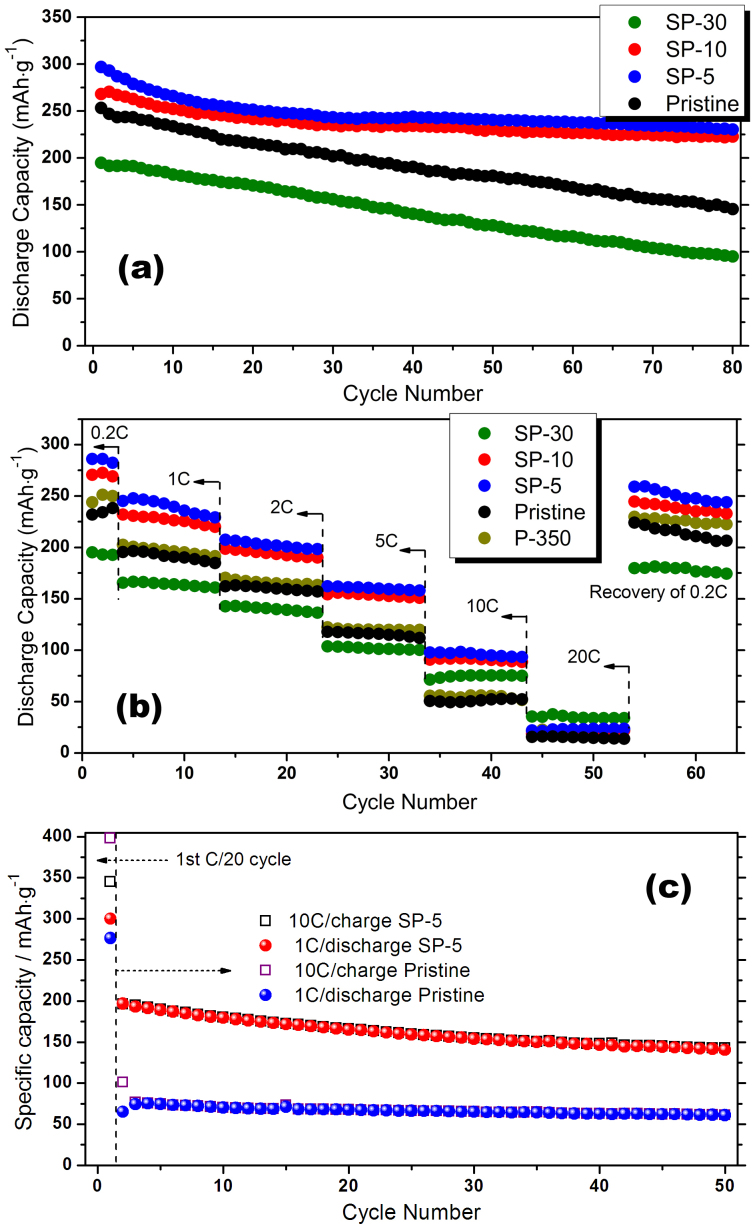
Cycle performance and rate capability of pristine, SP-5, SP-10 and SP-30 cathodes at different testing conditions: (a) 0.2 C, (b) at incremental C rates of 0.2, 1, 2, 5, 10 and 20 C (same current densities for charge/discharge) and (c) 10 C charge and 1 C discharge after an initial C/20 forming cycle. All the half batteries were cycled between 2.0 and 4.8 V at room temperature where 1 C stands for 250 mA·g^−1^.

Fig. 5 shows the cycle performance and rate capability of the LLNCM cathodes before and after modification. At a low rate of 0.2 C, both SP-5 and SP-10 exhibit a remarkable improvement of the ability of capacity retention compared to the pristine LLNCM, even though an initial capacity loss caused by structural evolution during first 10 cycles is still inevitable, as shown in Fig. 5(a). We propose that such improvement could be due to a protection effect induced by residual carbon on particle surfaces from HF attack during cycling. However, in the case of SP-30, a dominant factor switches while the serious spinel transformation unavoidably leads to more lattice disorder and dislocations in the particles which may further sacrifice the structural stability during cycling. Fig. 5(b) shows a continuous cycling result at incremental rates from 0.2 C to 20 C then recovering back to 0.2 C. As can be seen, the rate capability for both SP-5 and SP-10 has been enhanced before 20 C compared to the pristine one while SP-30 is the best at 20 C. Such contrast observation might be related to the higher diffusion capability of spinel-like phase than layered phase at an extremely high rate. To investigate the fast-charging ability, tests based on 10 C charge but 1 C discharge after an initial 0.05 C forming cycle were conducted on both pristine and SP-5 cathodes (Fig. 5(c)). Most surprisingly, SP-5 is capable of delivering almost 200 mAh·g^−1^ charge capacity and then stabilizing around 150 mAh·g^−1^ at such an extremely high rate as the pristine one only provides no more than 100 mAh·g^−1^ firstly and then stabilizing around 75 mAh·g^−1^. Such significant enhancement in rate capability is reasonably attributed to two reasons: one of which is the surface spinels; the other one is the coherent interaction induced by high temperature annealing between modified LLNCM particle and remained Super P particle to build better electron conduction network. Note that the widely-accepted poor rate performance for Li-rich layered cathodes is always ascribed to inferior electronic conductivity in conjunction with Mn^4+^ ions in Li_2_MnO_3_ component^4^,^15^,^29^. However, with spinel formation, the electronic conductivity could be enhanced to 10^−5^–10^−4^ S·cm^−1^ 30,31,32. On the other hand, the diffusion coefficients of Li (D_Li+_) for spinel LiMn_2_O_4_ are 10^−11^ to 10^−9^ cm^2^·s^-1^ 30, while the values of D_Li+_ for Li-rich layered cathodes are 10^−16^ to 10^−14^ cm^2^·s^−1^ 33,34. Therefore, no matter considering electronic or ionic conductivity of crystals, this surface transformation to spinel-like phases is always beneficial to improve rate capability. In addition, the charge/discharge curves with respect to Fig. 5(c) are shown in Fig. S6. It is important to note that the starting points of all charge curves at 10 C for SP-5 are around 3.0 V which means the spinel phase is still capable of contributing to fast ion flow, leading to lower polarization effects at surface. In contrast, these starting points in the pristine one are increasing from 3.0 V to almost 4.5 V which reflects a serious polarization induced by unmodified surface.

## Discussion

### Evidences of spinel formation on surface of modified LLNCM

**Figure 6:**
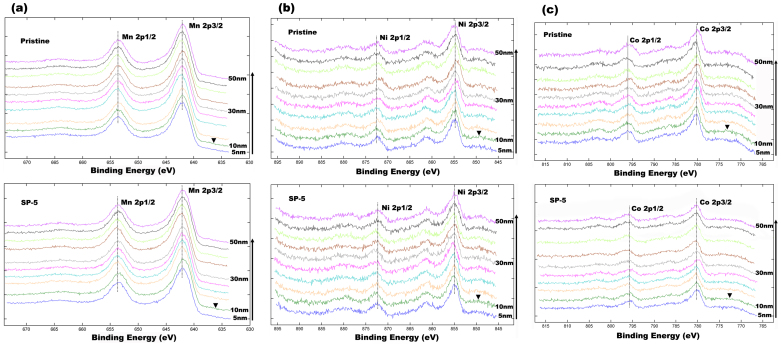
XPS spectra as a function of particle depth for pristine and SP-5 samples: (a) Mn 2p, (b) Ni 2p and (c) Co 2p. The ▾ symbol refers to the XPS spectra of 10 nm depth.

To determine the depth of spinels in the surface regions of LLNCM particles which are treated by Super P, a depth profiling XPS analysis on SP-5 sample was conducted. In this experiment, Ar^+^ ions were used to consecutively etch from particle surface to 50 nm in center with an etching rate around 5 nm·min^−1^. As shown in Fig. 6, the outermost surface XPS spectra were excluded because of contamination film and resultant energy shift, whereas all the other spectra with respect to Mn 2p, Ni 2p and Co 2p orbital have been compared to demonstrate the variations in chemical states as a function of depth. Fig. 6(a) shows the Mn 2p spectra. For SP-5 sample, a slight shift of peak toward lower binding energy for both 2p3/2 and 2p1/2 orbital could be observed, when comparing the profile of 10 nm with all other profiles at different depths. However, this observation has not been identified for the pristine sample, suggesting a variation in Mn oxidation state as a result of Super P treatment in this region. Since the binding energy of 2p orbital for trivalent Mn is lower than the one for tetravalent Mn, it is reasonable to deduce a higher amount of Mn^3+^ existing at 10 nm depth regions from particle surface compared to other regions inside particle, which strongly suggests a spinel-rich zone. Correspondingly, a similar peak shift of 10 nm profile for Ni 2p spectra toward higher binding energy is also observed in Fig. 6(b). It is likely that the increase in oxidation state of Ni is a compensation of deduction in oxidation state of Mn. However, there is no obvious change in oxidation state of Co as shown in Fig. 6(c). Based on these observations, it is reasonable to suggest that the spinels are rich at the depth of 10 nm from particle surface, which agrees well with HRTEM observation in Fig. 3D. In addition, a chemical composition analysis using XPS was also conducted and the results are shown in Fig. S7. It was found that the compositional ratio of Li relative to the sum of all transition metals (TM = Mn + Ni + Co) is reducing from 1.02 of pristine to 0.83, 0.82 and 0.4 respective to SP-5, SP-10 and SP-30. According to a basic concept that the ratios of Li: TM (x) in Li-rich layered and spinel structures are respectively based on the ranges of 1 < x < 2 and 1/2 < x < 1, it is also supportive for the spinel transformation on the surfaces of particle after Super P treatment.

**Figure 7:**
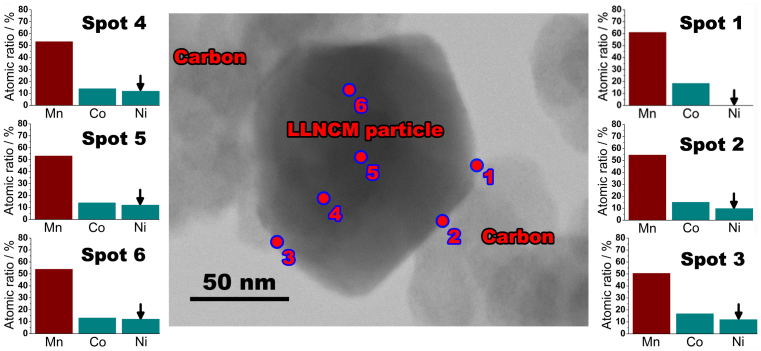
Typical TEM image of a SP-5 particle along with 6-spots EDS analysis results with respect to Mn, Ni, and Co represented by atomic ratios among them.

More evidences on the compositional variation of transition metals due to surface treatment could be found even at a nano scale. As detected by TEM-EDS using spot analysis, Fig. 7 shows a comparison of different nano-scaled regions in an as-prepared SP-5 particle to indicate the compositional changes through the surface to the inner part of such modified particle. Note that the electron beam for elemental analysis could be focalized to a quite small region with less than 10 nm diameter and the detection mode is transmitted. Therefore, the obtained values of relative composition of each transition metal are convincing and comparable. As shown in Fig. 7, the relative content comparing Ni to Co has almost no change with consideration of inner particle regions 4, 5, and 6, which is consistent with the original design Li_1.2_Mn_0.54_Ni_0.13_Co_0.13_O_2_. However, the Ni content relative to Co is apparently reduced on surface spots 2 and 3, further vanishing away in another surface spot 1. It is important to note that all the surface spots 1, 2 and 3 were chosen at the adherent locations between this particle and adjacent carbon spheres. Based on these observations, it is confident to deduce that such nano-scaled variation in transition metals of particular Ni is caused by the influence from adhesional carbon spheres, which could be taken as a supportive evidence of cation rearrangement during heat treatment. Due to the resolution limitation of such technique, it is hard to conclude the accurate chemical composition of the transformed phases on surface. However, based on a comparison between SP-5, pristine, LiMn_2_O_4_, and LiNi_0.5_Mn_1.5_O_4_ spinels (Fig. S8), their electrochemical behaviors suggest that the surface formed spinels are Ni/Co-contained spinels rather than accurate LiMn_2_O_4_ or LiNi_0.5_Mn_1.5_O_4_.

**Figure 8:**
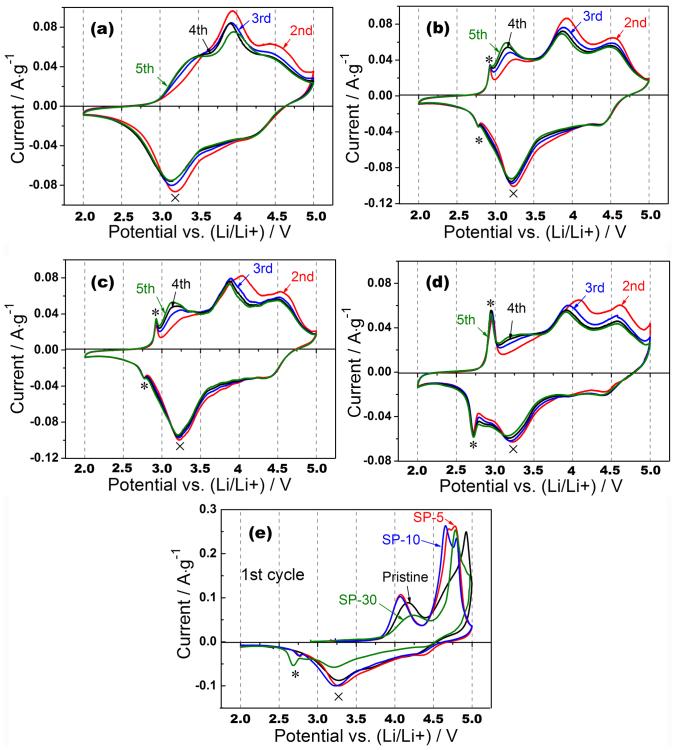
Cyclic voltammograms of the second to the fifth cycle of (a) pristine, (b) SP-5, (c) SP-10, (d) SP-30 and (e) the first cycle.

Fig. 8(e) shows the CV profiles of the first cycle of all LLNCM materials before and after treatment at a sweep rate of 0.1 mV·s^−1^. As can be seen, the initial anodic peaks of all the materials at about 4.1 V are due to the oxidation process of Ni^2+^/Co^3+^ to higher oxidation states^35^,^36^, while the intensity apparently varies because of the distinction of activated Ni and Co in different types of materials. The second anodic peaks located in the potential range between 4.5 and 5.0 V are normally associated with the oxygen release process from Li_2_MnO_3_-type component. Obviously, all the modified materials, i.e. SP-5, SP-10 and SP-30 exhibit shifted redox peaks to lower potentials than the counterpart in the pristine, which could be understood by reduced polarization effect caused by reduced internal resistance at a certain delithiated state of modified surface. During cathodic reactions in the first cycle, two broad peaks respectively located at around 4.3 and 3.2 V are reasonably regarded as the reduction processes of Ni^4+^/Co^3.6+^ and Mn^4+^ (marked as ×) in layered component^37^, while another cathodic peak only observed in SP-5, SP-10 and SP-30 around 2.7 V (marked as *) is believed to be due to insertion of Li accompanied with Mn^4+^ reduction in surface spinel component^38^. It suggests that such transformed surface spinels are able to be further lithiated when applied proper potential. Fig. 8(a), (b), (c) and (d) show the CV diagrams of the pristine, SP-5, SP-10 and SP-30 cathodes after first activation. A similar evolution route regarding CV profiles could be observed comparing the pristine with the modified samples. In detail, several broad peaks at 3.25, 3.9 and 4.6 V during anodic reactions along with 3.2 and 4.3 V cathodic reactions are regarded as a complex electrochemical delithiation/lithiation processes in both layered-type and transformed-spinel-type lattices. It is also interesting to point out that the totally different shapes of CV profiles comparing corresponding anodic/cathodic reactions may imply dissimilar mechanisms of Li transportation, which is still not clear at the current stage^38^. Based on Fig. 8, it is also noted that the redox pair within surface spinels (marked as *) exhibit increasing intensities from SP-5 to SP-30 through SP-10, indicating that the activated amount of the formed spinel components is increasing along with the increase of Super P.

**Figure 9:**
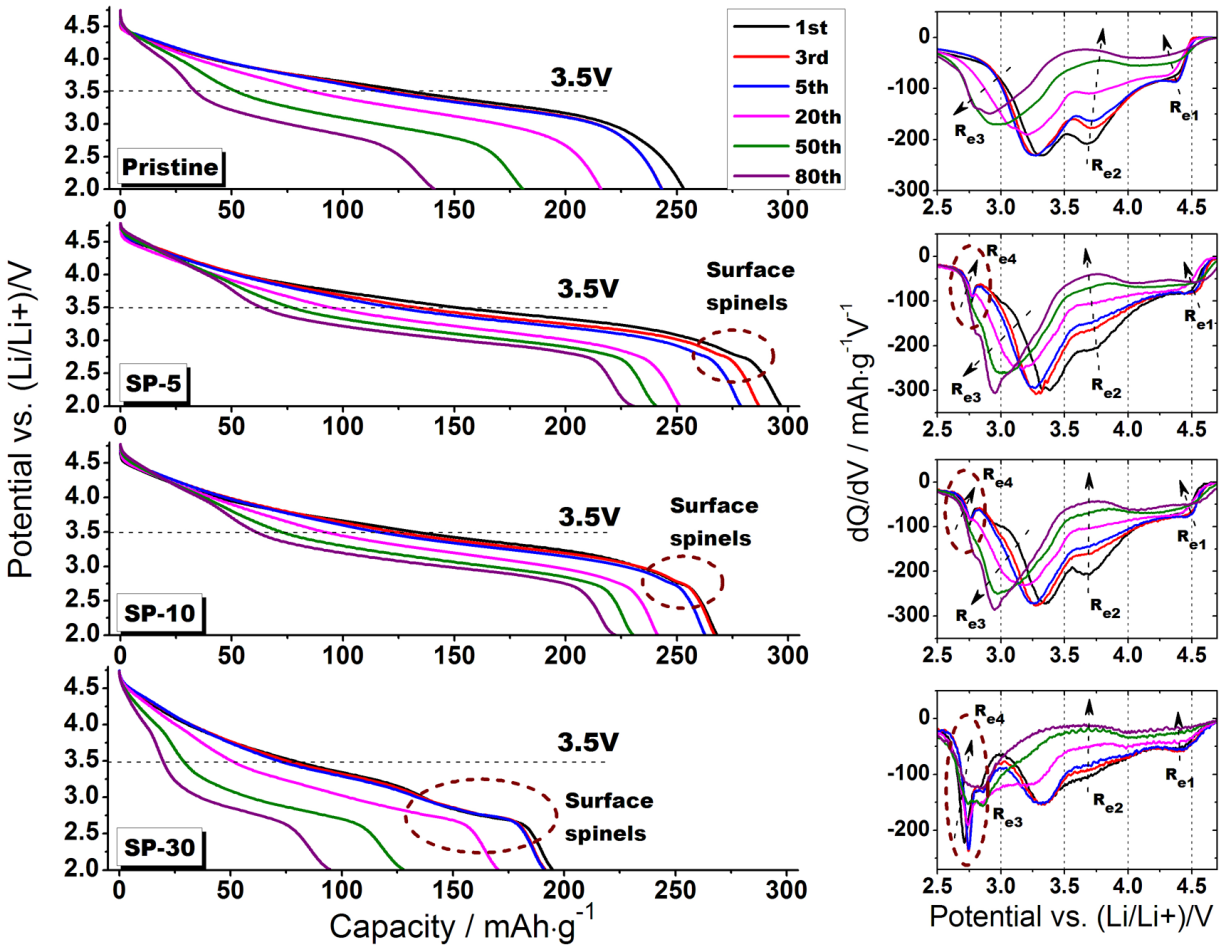
Discharge profiles with corresponding dQ/dV plots of pristine, SP-5, SP-10 and SP-30 materials. All batteries were cycled between 2.0 and 4.8 V at 50 mA·g^−1^ rate, 3.5 V is recognized as a knee point of different plateaus, and dash circles indicate the evolution process of surface spinels upon cycling.

To investigate the structural evolution in such pristine and modified cathode materials, discharge profiles in 0.2 C cycling tests as well as the corresponding dQ/dV plots of different stages of cycling, i.e. 1^st^, 3^rd^, 5^th^, 20^th^, 50^th^ and 80^th^ are shown in Fig. 9. The first observation from discharge profiles is that all the pristine and modified materials undergo a comparable shape change with the extent of cycling from 1^st^ to 80^th^, indicating a spinel transformation throughout particles during deep cycling since one discharge plateau gradually transform into two separated by the knee potential of 3.5 V^8^,^35^. It implies that the surface treatment through Super P may not help to suspend such phase transformation initiated by thermodynamic instability in the Li-rich group of layered materials. Another interesting observation from discharge profiles is that the 2.7 V plateaus associated with surface spinels are apparent in initial cycles (1^st^ and 3^rd^) but vanish in extended cycles (50^th^ and 80^th^), which may be explained by transition metals migration and induced lattice rearrangement as a result of interaction between surface regions and inner particle during cycling^39^,^40^. dQ/dV plots of the pristine sample clearly show three individual reduction processes during discharge, i.e. R_e1_ related to Li occupation within tetrahedral sites according to theoretical and NMR studies^37^, R_e2_ related to Li occupation within octahedral sites accompanied with Ni/Co redox and R_e3_ related to Li occupation within octahedral sites associated with Mn redox. As clearly shown by dash arrows, R_e1_ and R_e2_ contributions to discharge capacity are gradually decreasing upon cycles as the contribution due to R_e3_ redox increases step by step, although moving towards lower potential as a result of transformation from layered structure to spinel-like structure. It is important to note that a similar structural evolution process in consideration of R_e1_, R_e2_ and R_e3_ for both pristine and SP-5 (SP-10) materials took place but with variations in redox intensities. Specifically, the intensity of R_e3_ redox becomes higher and higher upon cycling in SP-5 (SP-10) which indicates that the activated Mn^4+^/Mn^3+^ redox in corresponding structure has more contribution in capacity than the pristine one does. On the other hand, the R_e4_ redox related to surface spinels is gradually vanishing in all the modified materials which is consistent with the observation from discharge profiles. Regarding SP-30, because of the significant transformation effect led by Super P treatment, the overlapping between R_e3_ and R_e4_ redox makes it hard to distinguish them from each other, while they apparently merge into one at the end of 80 cycles. As a matter of fact, the end plots of dQ/dV for 80^th^ cycle comparing all of the samples are quite similar, indicating that no matter modified or pristine structures tend to reach a similar final member as a result of intrinsic thermodynamic force led by vacancies and defects.

**Figure 10:**
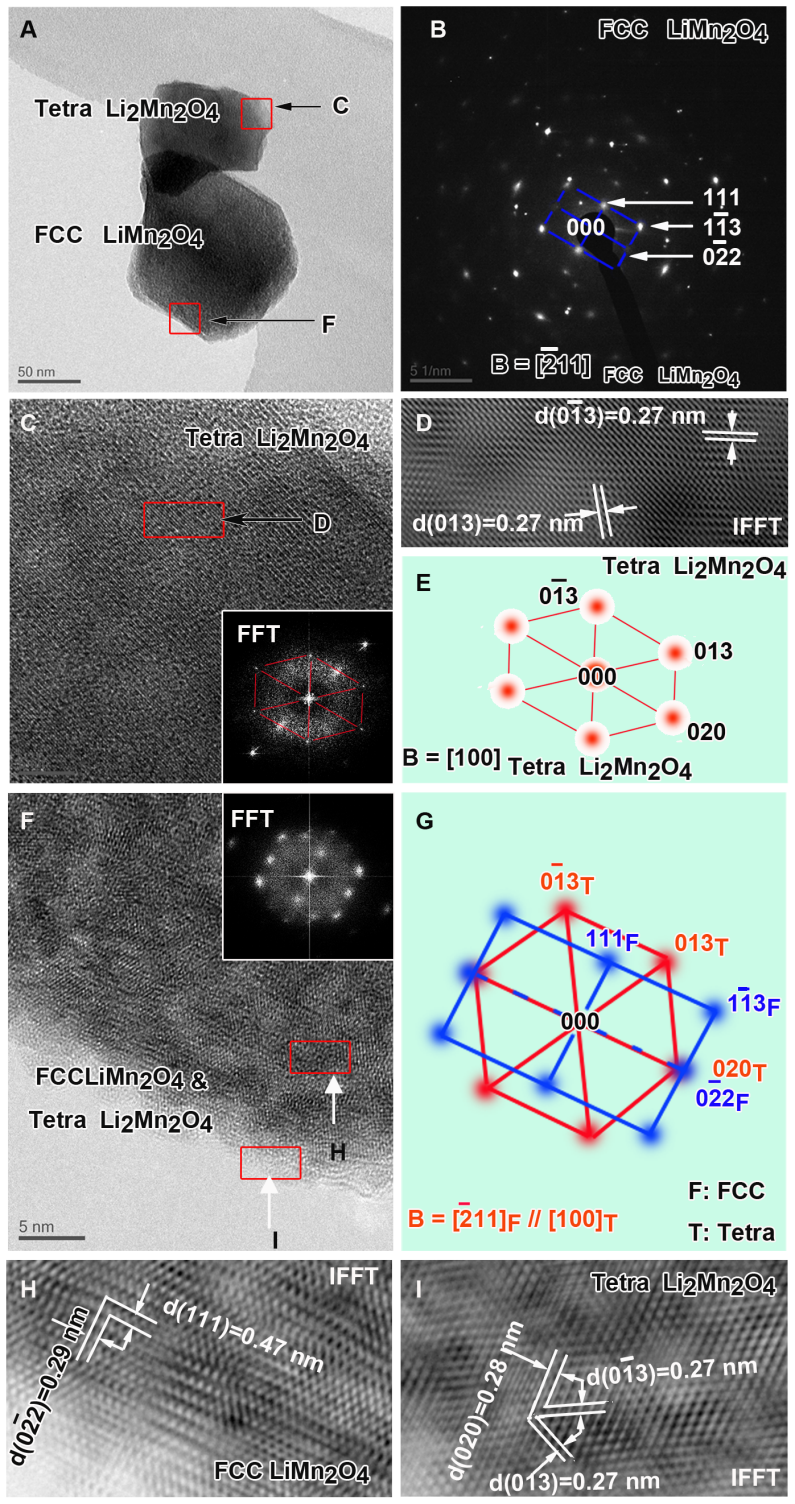
TEM investigation of SP-5 sample after 20 cycles.(A). Conventional TEM bright field image. The red squares have been extensively imaged in Panel C and F. (B). The selected electron diffraction (EDP) of Panel A. It could be indexed with FCC LiMn_2_O_4_, while other diffraction spots are from tetragonal Li_2_Mn_2_O_4_. (C). HRTEM image from the upper square area in Panel A. Inset is a Fast Fourier Transformation (FFT) image which could be indexed as [100] zone axis of tetragonal Li_2_Mn_2_O_4_ phase (Panel E). It is also confirmed by an inverse FFT (IFFT) image (Panel D) with two lattice fringes of plane (013) and (0–13) (d-spacing: 0.27 nm). (F). HRTEM image from the lower red square area in Panel A with inset of FFT image. From FFT image it could be deduced that the main phase is FCC LiMn_2_O_4_ (blue pattern in Panel G) doped with many nano-sized tetragonal Li_2_Mn_2_O_4_ phase (red pattern in Panel G). (G). Composited EDPs reveal the mutual orientation relationship between FCC LiMn_2_O_4_ and tetragonal Li_2_Mn_2_O_4_ phases: [-211]_F_//[100]_T_; (0-22)_F_//(020)_T_; (111)_F_//(006)_T_. H and I are IFFT images providing lattice fringes for both phases. Their lattice spacing are all consistent with those calculated results.

To show the nature of the particle surface after 20 cycles of electrochemical charge/discharge, SP-5 sample was cycled at 10 C charge and 1 C discharge condition after a first C/20 forming cycle (same condition as in Fig. 5(c)). Then the SP-5 particle was characterized using HRTEM. As shown in Fig. 10, several interesting observations could be identified: (a) Two types of structures, i.e. face-centered cubic spinel and tetragonal spinel are apparent whereas the original layered phase is absent because of electrochemical cycling. (b) The cubic spinel is reasonably derived from the transformed Li_2_MnO_3_-type structure, which has been evidently observed in previous reports^8^,^41^. On the other hand, the tetragonal spinel is likely to be associated with initially-formed cubic spinel which is ascribed to Super P treatment, because it locates at outermost surface of particle as indicated in Fig. 10F. Note that the particle is at a fully-discharged state of 2 V, which means one mole of Li could be further intercalated into cubic LiM_2_O_4_ spinel to form tetragonal Li_2_M_2_O_4_ spinel (M indicates Mn, trace doped Ni and Co)^42^,^43^. (c) A larger amount of tetragonal phase at upper particle surface (Fig. 10C) compared to the lower one is possibly due to more significant transformation in this region during Super P treatment. Based on the above observations, it is reasonable to deduce that the surface phase in form of tetragonal spinel play a critical role in fast extraction of Li and electrons for the following charge process.

### Benefits from surface spinels according to EIS

**Figure 11:**
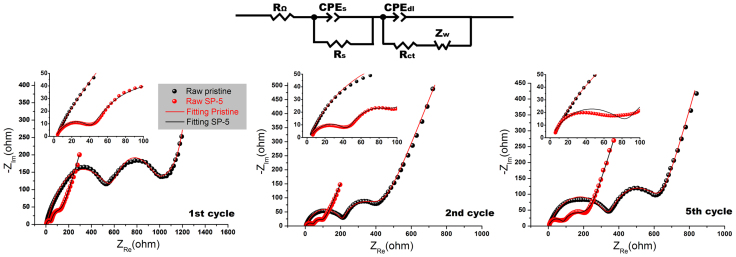
EIS spectra of pristine and modified SP-5 materials with respect to 1^st^, 2^th^ and 5^th^ cycles at a same state of discharge (3.5 V). The equivalent circuit used for spectra fitting is also shown.

Fig. 11 compares the EIS spectra of the pristine and the modified SP-5 samples at different states of cycling, i.e. 1^st^, 2^nd^ and 5^th^. An equivalent circuit^44^ was applied to fit the raw data from EIS measurements to obtain the accurate values of different resistances. In this equivalent circuit, R_s_ is the resistance of ion diffusion in the region of the surface layer of particle, R_ct_ represents the charge transfer resistance, and Z_w_ is the Warburg impedance for describing Li diffusion in the bulk regions of material. In our case, both R_s_ and R_ct_ resistances upon cycling are under consideration since they reflect surface features. As summarized in Table 3, the SP-5 sample always reveals much lower values compared to the ones for pristine sample which can be ascribed to three reasons: (a) the surface spinels for SP-5 particles possess 3-D channels for Li diffusion while the original layered surface only has 2-D; (b) the surface treatment also leads to a collapse of Ni segregation which could suppress negative effects on fast transportation of Li between terminated surfaces and the bulk^12^, as supported by Fig. 7; (c) the enhanced contact between the residual Super P spheres and particle surfaces may provide better surface conductivity leading to significantly reduced R_ct_. In fact, such observations on EIS are consistent with the results obtained from electrochemical testing.

In conclusion, to obtain both improved first coloumbic efficiency and fast charging ability of Li(Li_0.2_Mn_0.54_Ni_0.13_Co_0.13_)O_2_ cathode material while retaining the high specific capacity of such Li-rich layered group of materials, a simple strategy of surface treatment led by Super P (carbon black) was introduced in this work. The modified SP-5 cathode material is not only capable of delivering as high as 200 mAh·g^−1^ charge capacity at an extremely high rate of 10 C (2500 mA·g^−1^), but also improve the first coloumbic efficiency from 78% to 93.4% at C/20. According to HRTEM, TEM-EDS, XPS and EIS results, such improvements on electrochemical performance could be explained by surface transformation from original Li_2_MnO_3_-type to spinel-type structure due to the interaction between particle surfaces and carbon spheres during post annealing process. The structural compatibility between layered and transformed spinels in view of unique orientations ([201]*_layered_* and [41-1]*_spinel_*) was also observed using HRTEM. Most importantly, this is the first time to report such unique phenomenon that besides acid environment, which could lead to leaching of Li and subsequent phase transition in layered materials, the carbon is also able to transform Li_2_MnO_3_-type of cathode materials. This transformation might be understood by the reductive environment provided by carbon on Mn^4+^ at an increased temperature. Although the intrinsic reasons are unclear, the facility and effectiveness still make this modification strategy promising to be applied at industrial level when the enhanced rate performance is needed in Li-rich Mn-based layered cathodes.

## Methods

### Preparation and modification

The pristine layered Li(Li_0.2_Mn_0.54_Ni_0.13_Co_0.13_)O_2_ (LLNCM) compound was synthesized using a traditional sol-gel method assisted by spray-drying machine (YC-015, Shanghai Politech Instrument & Equipment Co. Ltd). The details are described in another report^45^. In a typical treatment, the as-prepared LLNCM powder (1 g) was mixed with different weight ratios of Super P in the solution of NMP (50 mL) with help of ultrasonic dispersion for 30 min followed by constantly stirring for overnight at room temperature. According to the different weight ratios applied to treat LLNCM, 5 wt.%, 10 wt.% and 30 wt.% treated samples are designated as SP-5, SP-10 and SP-30, respectively. After stirring, the blend was dried at 130°C for 12 h in air to evaporate NMP solvent. Then, the obtained powder was heated at 350°C for 2 h with 5°C·min^−1^ heating rate in air. To study the influence of heat treatment without Super P, pristine LLNCM was post treated at 350°C for 2 h and termed as P-350.

### Structure characterizations

The crystallographic features of the synthesized LLNCM were characterized by powder X-ray diffraction (Shimadzu XRD-6000 Cu-Kα radiation, λ = 1.5418 Å). The diffraction data were collected at a scan rate of 0.5° min^−1^ in the 2θ range of 10–80°. Particle morphologies and detailed lattice information were revealed by a scanning electron microscope (Fe-SEM) (S-4300 Shimadzu, 15 kV) and a high-resolution transmission electron microscope (HRTEM) (JEOL 2200FS, operating at 200 kV). Elemental analysis of particle was carried out using an Oxford INCA Energy EDX System equipped with a JEOL-JEM 3010 TEM. The solid-state chemistry of the pristine and treated powders were investigated by X-ray photoelectron spectroscopy (XPS, PHI Quantera II XPS Scanning Microprobe). Ar^+^ etching was applied to determine the depth profiles of transition metals Mn, Ni and Co from surface to 50 nm inside particles. The etching rate was set to be 5 nm/step. Standard binding energies of XPS peaks were referred to literature values^46^,^47^. Thermogravimetric analysis (TGA) was carried out on both pristine and treated samples in air at a heating rate of 5°C·min^−1^ from room temperature to 800°C using DTG-60H Shimadzu.

### Electrochemical measurements

All the electrochemical cycling tests were performed using coin cells (CR-2016). The electrode slurries were firstly prepared by mixing as-prepared materials, carbon black and polyvinylidene fluoride (PVDF) in a weight ratio of 80:10:10 within n-methyl-2-pyrrolidone (NMP) solution. Then the slurries were constantly stirred for overnight to obtain uniform dispersion of all contents. Finally the slurries were pasted onto an aluminum foils which were dried at 120°C for overnight before assembling. The typical loading density of prepared electrodes are about 4–5 mg·cm^−2^. A half cell was assembly using the prepared electrode as a cathode, two pieces of separator (Celgard 2500), pure Li foil as the anode, and electrolyte (1 M LiPF6 in EC: DEC = 1:1 organic solutions). Maccor 4 and Neware Battery Test Station were used to perform galvanic charge and discharge testing in which all of them were conducted in a voltage range of 2.0–4.8 V vs. Li^0^/Li^+^ (where the current density at 1 C is 250 mA·g^−1^) at room temperature. Cyclic voltammetry was also applied with a scan rate of 0.1 mV·s^−1^ between 2.0 and 5.0 V vs. Li^0^/Li^+^. Electrochemical impedance spectroscopy (EIS) was conducted using a Solartron 1260 + 1287 System at frequency range from 100 kHz to 0.001 Hz with an AC voltage amplitude of 5 mV. For each state of cycling, all the samples were charged to the cutoff voltage of 4.8 V and then discharged to the same state of 3.5 V holding for another 3 h before the EIS measurement. Plots of dQ/dV vs. voltage were calculated based on the testing data of charge/discharge.

## Author Contributions

B.S., P.X. and L.L. conceived the experiments and B.S. completed all of the works except for HRTEM. H.L. and Z.L. accomplished the HRTEM experiments in addition to the analysis. B.S., M.O.L. and L.L. wrote the initial manuscript which was approved by all the authors.

## Supplementary Material

Supplementary Information High rate capability caused by surface cubic spinels in Li-rich layer-structured cathodes for Li-ion batteries

## Figures and Tables

**Table 1:** Lattice parameters of Li(Li_0.2_Mn_0.54_Co_0.13_Ni_0.13_)O_2_ before and after surface treatment with various amounts of Super P followed by annealing at 350°C

	a (Å)	b (Å)	c (Å)	c/a	Vol (Å^3^)	R_wp_
Pristine	2.86630(8)	2.86630(8)	14.3121(8)	4.993	101.831(9)	9.5
SP-5	2.86657(8)	2.86657(8)	14.3101(8)	4.992	101.836(9)	8.1
SP-10	2.86704(9)	2.86704(9)	14.3097(9)	4.991	101.873(10)	9.0
SP-30	2.86741(9)	2.86741(9)	14.3069(9)	4.989	101.872(10)	11.7

**Table 2:** First charge/discharge capacities and corresponding coulombic efficiency of pristine, SP-5, SP-10 and SP-30 cathodes when cycled between 2.0 and 4.8 V at 12.5 mA·g^−1^ (C/20)

	Charge capacity (mAh·g^−1^)	Discharge capacity (mAh·g^−1^)	First coulombic efficiency (%)
Pristine	332	259	78
SP-5	333	311	93.4
SP-10	325	286	88
SP-30	285	222	77.9

**Table 3:** Fitting values of R_s_ and R_ct_ of the pristine and the modified SP-5 materials at different states of cycling

	1^st^ cycle		2^nd^ cycle		5^th^ cycle	
	Pristine	SP-5	Pristine	SP-5	Pristine	SP-5
R_s_ (ohm)	590	41	215	45	352	89
R_ct_ (ohm)	410	94	122	42	268	70
