# Supplementary material for: High rate capability caused by surface cubic spinels in Li-rich layer-structured cathodes for Li-ion batteries
Source: Sci Rep. 2013 Oct 31;3:3094. doi: 10.1038/srep03094 (PMC3813932; doi:10.1038/srep03094)
Supplement: Supplementary Information — High rate capability caused by surface cubic spinels in Li-rich layer-structured cathodes for Li-ion batteries [file srep03094-s1.pdf]

Supporting Information

High rate capability caused by surface cubic spinels in Li-rich layer-structured cathodes for Li-ion batteries

Bohang Song[1], Hongwei Liu[2], Zongwen Liu[2], Pengfei Xiao[1], Man On Lai[1], Li Lu[1,*]

[1] Materials Science Group, Department of Mechanical Engineering

National University of Singapore, Singapore

[2] School of Chemical and Bimolecular Engineering

The University of Sydney, Australia

[*] Correspondence to luli@nus.edu.sg

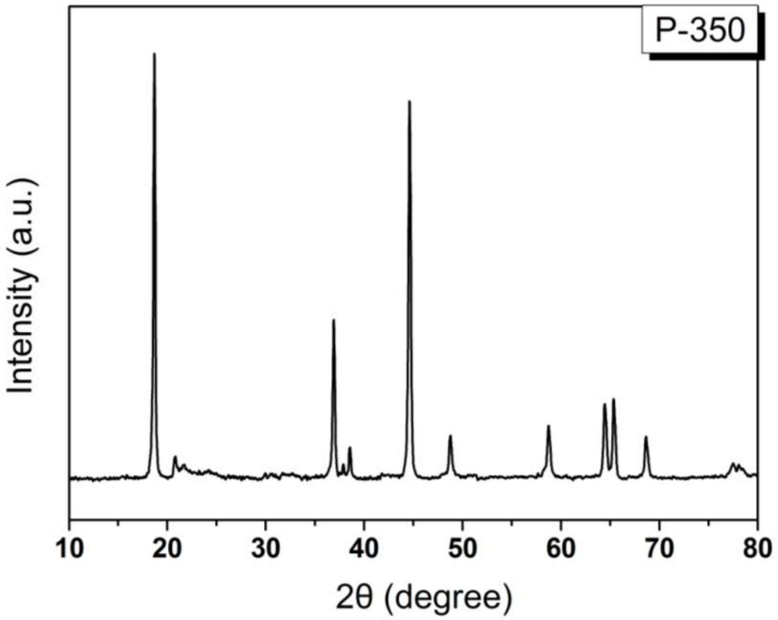

Fig. S1 XRD pattern of the P-350 powder.

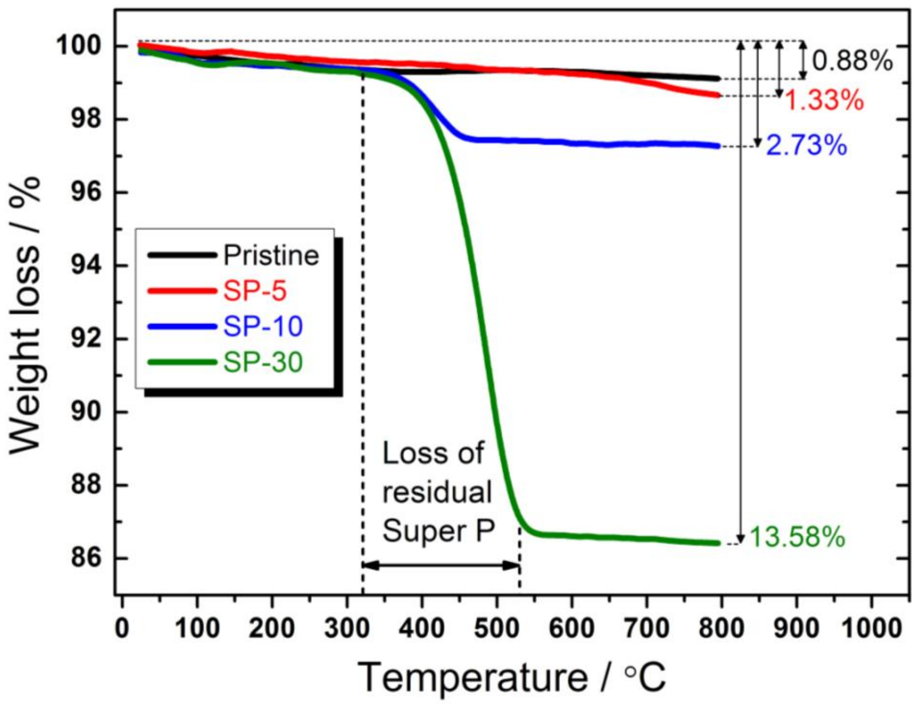

Fig. S2 TGA plots of pristine, SP-5, SP-10 and SP-30 powders. (Heating rate = 5 °C·min^{-1} under an air atmosphere).

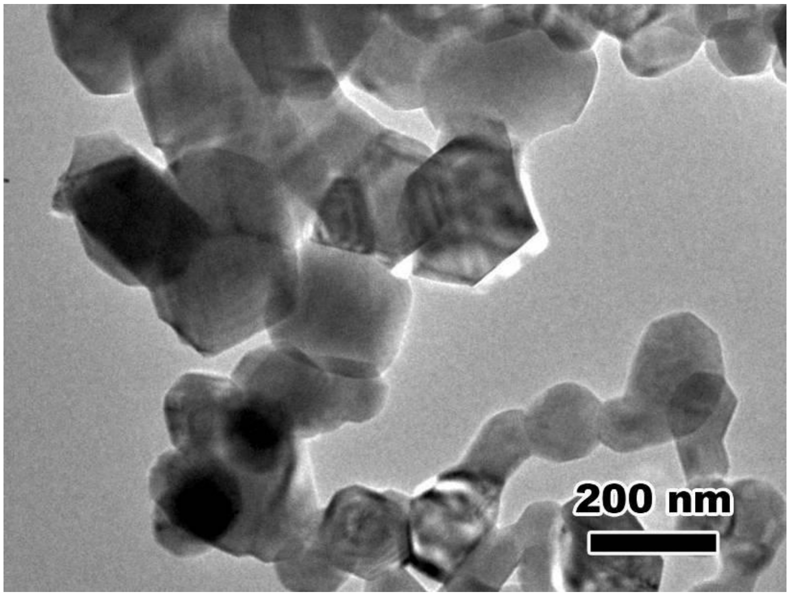

Fig. S3 Bright field TEM image of the pristine $Li(Li_{0.2}Mn_{0.54}Ni_{0.13}Co_{0.13})O_2$ particles.

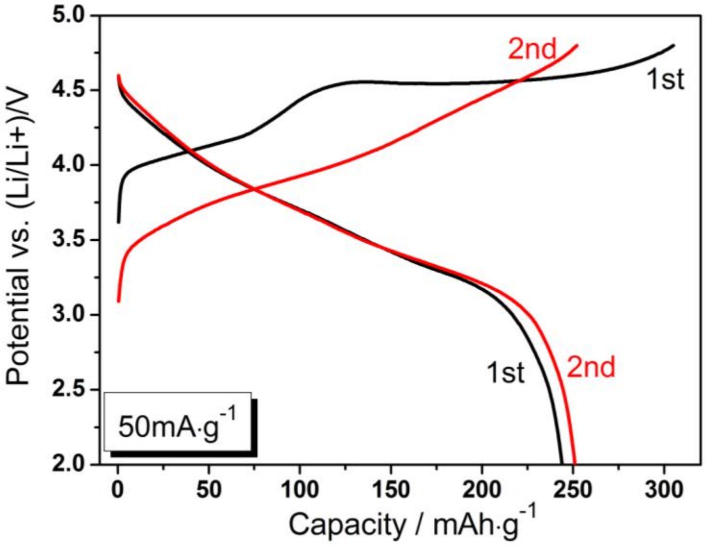

Fig. S4 Charge/discharge curves of first two cycles of P-350 electrode cycled at 50 mA g^{-1}.

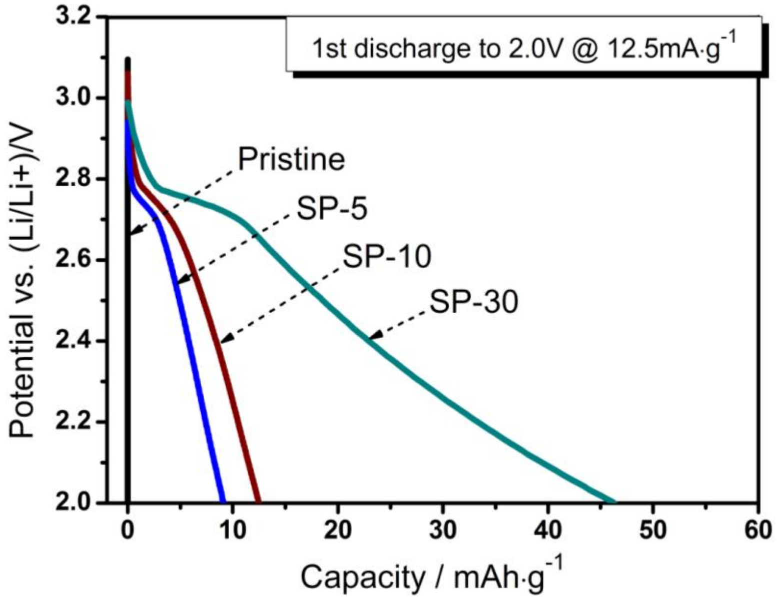

Fig. S5 First discharge curves from OCP to 2.0 V started from fresh cells containing these cathodes at 12.5 mA g^{-1}.

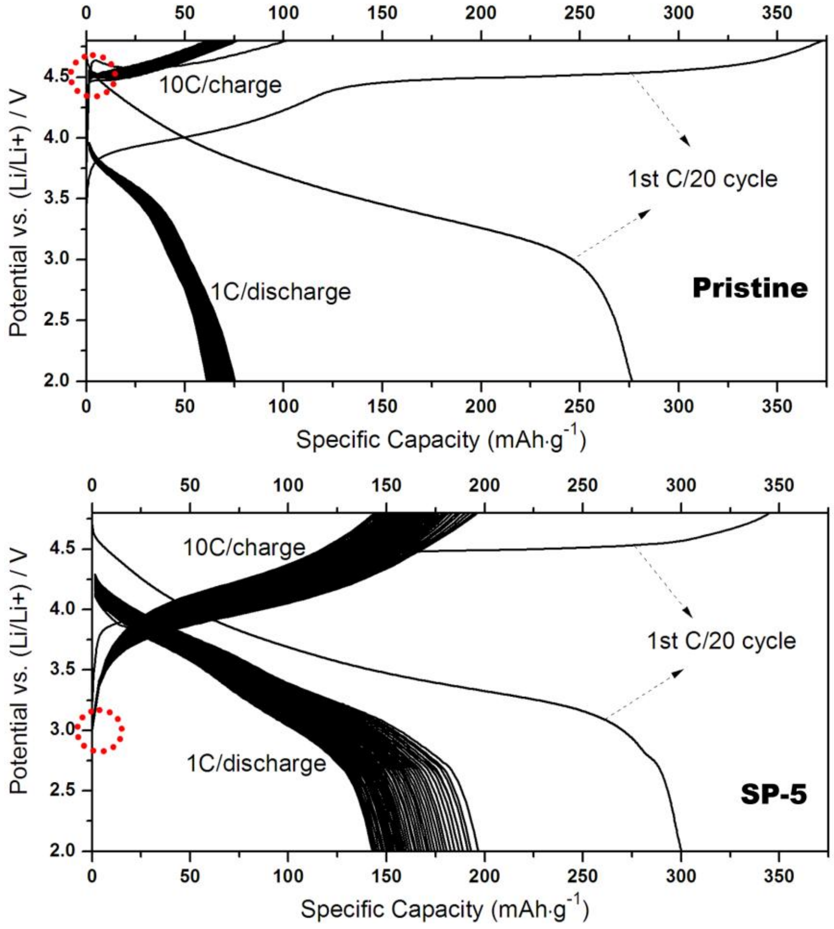

Fig. S6 10C-charge/1C-discharge curves after an initial forming cycle of both pristine and SP-5 cathodes cycled between 2.0 and 4.8 V where 1 C corresponds to 250 mA·g^{-1}.

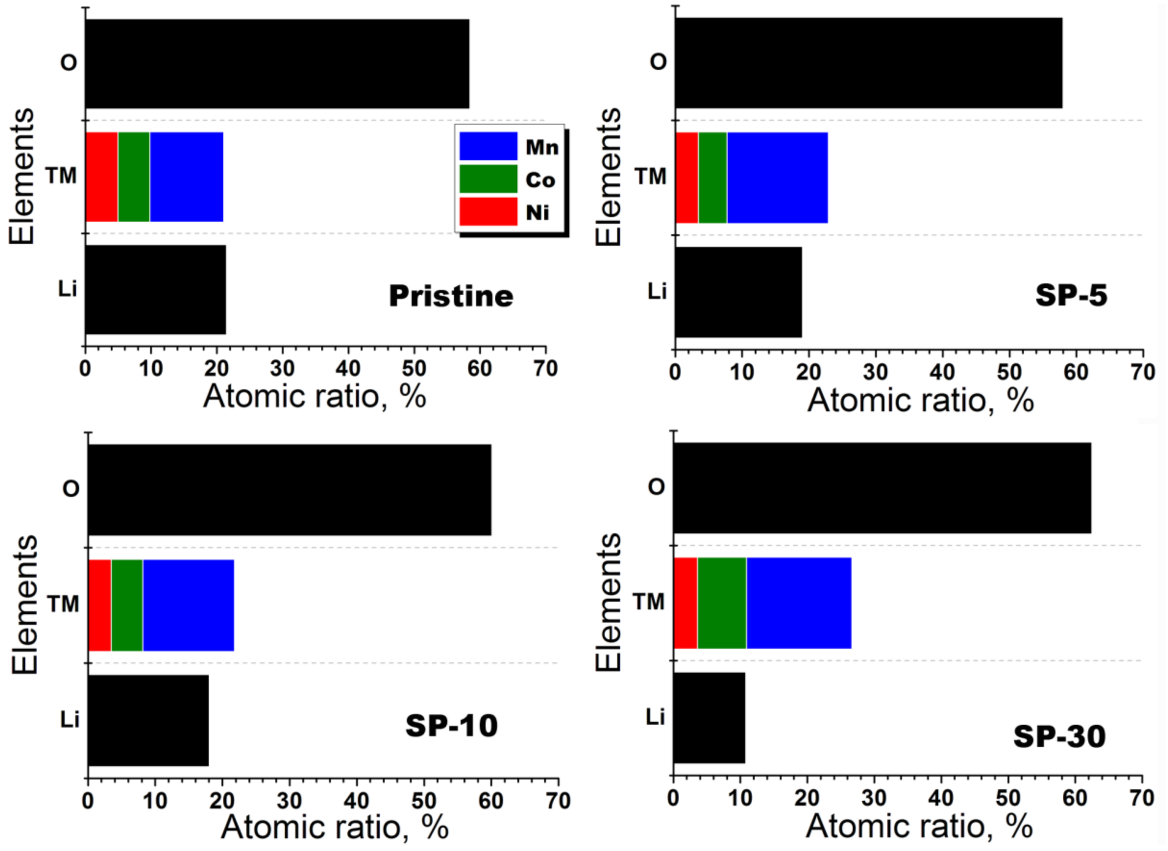

Fig. S7 Surface analysis of chemical composition using XPS technique with respect to pristine, SP-5, SP-10 and SP-30 samples.

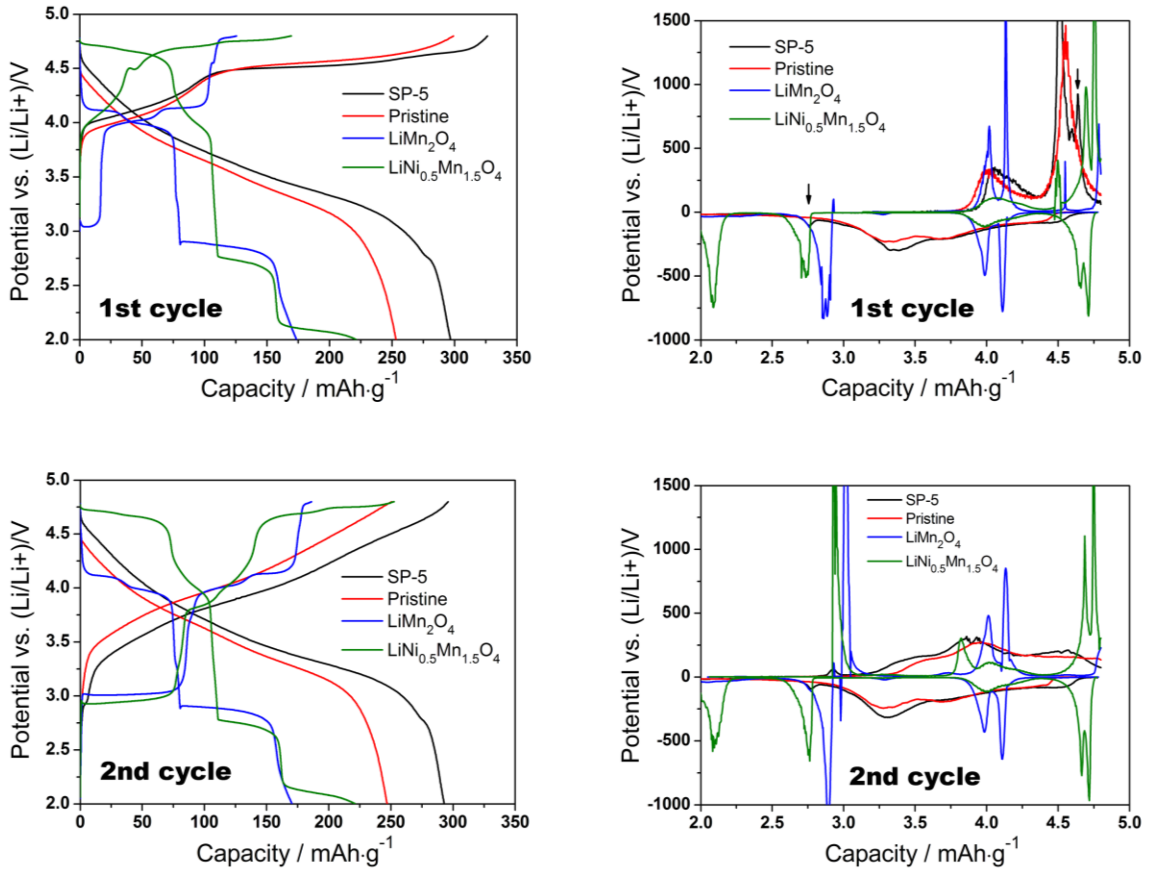

Fig. S8 Charge/discharge curves and corresponding dQ/dV plots of SP-5, pristine, $LiMn_2O_4$ and $LiNi_{0.5}Mn_{1.5}O_4$ cycled between 2.0 and 4.8 V at 50 mA g^{-1}. As can be seen from dQ/dV plots of first cycle, although the additional peak (marked as ↓) in charging process of SP-5 located at 4.64 V is comparable to the peak (4.70 V) only observed for $LiNi_{0.5}Mn_{1.5}O_4$ sample, they are not located at the exactly same position (two electrodes possess similar loading density). Besides, upon first discharging process, two peaks (4.65 and 4.72 V) observed for $LiNi_{0.5}Mn_{1.5}O_4$ are both missing for SP-5 sample, indicating different intercalation mechanisms in this voltage region. Additionally, the newly-formed peak (2.76 V) upon discharge for SP-5 sample is in between 2.72 V ($LiMn_2O_4$) and 2.82 V ($LiNi_{0.5}Mn_{1.5}O_4$), also indicating spinel behavior rather than exact $LiMn_2O_4$ or $LiNi_{0.5}Mn_{1.5}O_4$. The second cycle also suggests similar conclusion.
